# Supplementary material for: Bovine tuberculosis model validation against a field study of badger vaccination with selective culling
Source: PLoS One. 2025 Jul 21;20(7):e0320830. doi: 10.1371/journal.pone.0320830 (PMC12279149; doi:10.1371/journal.pone.0320830)
Supplement: S1 Table — (DOCX) [file pone.0320830.s001.docx]

**Table S1- Input model parameters.**

| **Parameter** | **Value** | **Parameter description** | **Reference** |
| --- | --- | --- | --- |
| **Arena parameters** | | | |
| **Grid size** | 20km^2^ by 20km^2^ | Area of total landscape. Landscape is divided into grid squares of 200m by 200m | Model assumption |
| **Core size** | 100km^2^ | Area of control | Matched to study |
| **Badgers** | | | |
| **Number of social groups in core** | 85 |  | Model assumption |
| **Group carrying capacity** | Average: 3  Range: 2-4 |  | Fitted to give mean group size approximating mean estimate from Woodchester park of 8.6 [1]. |
| **Seeding- average number at start of simulation** | Juvenile male: 0.8  Yearling male: 0.6  Adult male: 1.2  Juvenile female: 0.9  Yearling female: 0.7  Adult female: 2.1 |  | Matched to demographic distribution at Woodchester park at start of January |
| **Breeding probability** | 1^st^ female: 0.85  2^nd^ female: 0.4  3^rd^ female: 0.4  4^th^ female: 0.4 |  | Calibrated based on proportion of females reproducing from Woodchester park population [1] |
| **Litter size probability** | 1 cub: 0.08  2 cubs: 0.26  3 cubs: 0.77  4 cubs: 0.95 | Probability of each potential litter size. If p>0.95, litter size is 5. | Mean of 2.94 [2] |
| **Pre-emergence cub mortality** | 0.24 |  | [1] |
| **Mortality** | Male: TB-free: 0.0637  Female: TB-free: 0.05    Male: ELISA-positive: 0.0908  Female: ELISA-positive:  0.0513    Male: single-site excretor:  0.1168  Female: single-site excretor:  0.0479    Male: multi-site excretor:  0.2831  Female: multi-site excretor:  0.1461 | Male: TB-free: 0.07485  Female: TB-free: 0.058    Male: ELISA-positive: 0.107  Female: ELISA-positive:  0.0603    Male: single-site excretor:  0.1372  Female: single-site excretor:  0.0563    Male: multi-site excretor:  0.3326  Female: multi-site excretor:  0.1717 | Estimated from capture-mark-recapture data set of Woodchester park population [3]. Probabilities are adjusted to be linearly inversely proportional to group size. |
| **Dispersal probability** | Male: 0.00939  Female: 0.000834 | Probability of dispersal per two-month period. Equivalent to annual probability of 0.055 and 0.005. | [4] |
| **Dispersal receiver threshold** | 1 | Difference in number of one sex between groups that will trigger inwards movement |  |
| **Dispersal difference threshold** | 3 | Smallest difference between a group and its neighbour that will still trigger an inwards move |  |
| **Control parameters** | | |  |
| **Control months** | August (8) and October (10) |  |  |
| **Participation probability** | 0.94 | Proportion of landowners within core participating in control | F. Menzies pers. comm. |
| **Trapping efficacy** | 0.54 |  |  |
| **Control proportion parameters** | |  |  |
| **July and August trapping parameters** | 0.543 |  | Estimated |
| **Badger vaccine efficacy** | 0.6 |  | Estimated |
| **DPP sensitivity** | 0.63 |  | [6] |
| **DPP specificity** | 0.98 |  | [6] |
| **Farm parameters** | | |  |
| **Farm density** | 2.18 farms per km^2^ |  | From study |

1. Rogers, L.M.; Cheeseman, C.L.; Mallinson, P.J.; Clifton-Hadley, R. The demography of a high-density badger (*Meles meles*) population in the west of England. *J. Zool.* **1997**, *242*, 705-728, doi:<https://doi.org/>.

2. Neal, E.; Cheeseman, C. *Badgers*; T & AD Poyser: London, UK, 1996; p. 271.

3. Graham, J.; Smith, G.C.; Delahay, R.J.; Bailey, T.; McDonald, R.A.; Hodgson, D. Multi-state modelling reveals sex-dependent transmission, progression and severity of tuberculosis in wild badgers. *Epidemiol. Infect.* **2013**, *141*, 1429-1436, doi:<https://doi.org/10.1017/S0950268812003019>.

4. Rogers, L.M.; Delahay, R.; Cheeseman, C.L.; Langton, S.; Smith, G.C.; Clifton-Hadley, R.S. Movement of badgers (Meles meles) in a high density population: individual, population and disease effects. *Proc. R. Soc. B* **1998**, *265*, 1269-1276, doi:<https://doi.org/10.1098/rspb.1998.0429>.

5. Menzies, F.D.; McCormick, C.M.; O'Hagan, M.J.H.; Collins, S.F.; McEwan, J.; McGeown, C.F.; McHugh, G.E.; Hart, C.D.; Stringer, L.A.; Molloy, C.; et al. Test and vaccinate or remove: Methodology and preliminary results from a badger intervention research project. *Vet. Rec.* **2021**, e248, doi:<https://doi.org/10.1002/vetr.248>.

6. Arnold, M.E.; Courcier, E.A.; Stringer, L.A.; McCormick, C.M.; Pascual-Linaza, A.V.; Collins, S.F.; Trimble, N.A.; Ford, T.; Thompson, S.; Corbett, D.; et al. A Bayesian analysis of a Test and Vaccinate or Remove study to control bovine tuberculosis in badgers (*Meles meles*). *PLoS One* **2021**, *16*, e0246141, doi:<https://doi.org/1371/journal.pone.0246141>.
