# Supplementary material for: Bovine tuberculosis model validation against a field study of badger vaccination with selective culling
Source: PLoS One. 2025 Jul 21;20(7):e0320830. doi: 10.1371/journal.pone.0320830 (PMC12279149; doi:10.1371/journal.pone.0320830)
Supplement: S2 File — (DOCX) [file pone.0320830.s002.docx]

**Badger-TB TVR Model ODD description**

The model description follows the ODD (Overview, Design concepts, Details) protocol for describing individual- and agent-based models (Grimm et al., 2006), as updated by Grimm et al. (2020). The model is implemented in the Python3 programming language (Van Rossum and Drake, 2009).

**1. Purpose**

The model, known as TBi, was created to provide advice for the formulation of policy decisions around the issue of bovine tuberculosis in wild badgers. The model simulates population and disease dynamics in an undisturbed badger population and its response to management, allowing the effectiveness of proposed management strategies at reducing disease to be assessed before operational use (Smith et al., 1997; Smith et al., 2001a; Smith et al., 2001b; Wilkinson et al., 2004; Wilkinson et al., 2009). The patterns used as criteria for evaluating the model’s purpose comprise the size of the badger population and the number of infected badgers before, during and after management operations, and their spatial distribution in the model arena.

In the version presented here, it is used to understand the potential influence of a TVR approach (Trap, Vaccinate, Remove) on the prevalence of TB in a representation of the Northern Ireland badger population.

**2. Entities, state variables, and scales**

The individual based model (IBM) represents one type of agent, individual badgers. They are characterised by the state variables of:

1. ID (Numeric ID)
2. Sex (Male/Female)
3. Age (Cub/Juvenile/Adult)
4. Disease state (SEIR)
5. Group (Numeric ID)
6. Vaccination status
7. Dispersal status

Badgers belong to social groups (collective entities), but they may move from one group to another. There is a single social group within each badger territory, and each social group has a set of neighbouring groups determined by the spatial distribution of territories.

The model arena comprises a spatial grid of 100 x 100 cells of 4ha, i.e. 400 km^2^, which provides sufficient resolution to ensure a realistic representation of each badger territory’s neighbours. The model’s spatial entities are badger social group territories and farms comprising a certain number of contiguous grid cells. The model is toroidal so territories on one edge of the arena neighbour those on the opposite edge. Badger territories are characterised by the location of the social group’s main sett and a carrying capacity regulating the size of the occupying social group. Farms are characterised by whether they are participating in disease management operations, but not any further simulation of farm characteristics (e.g. cattle). Badger territories are classified as being part of a central management zone of defined size, within a surrounding ring approximately two social groups wide where the effects of social perturbation are expected, or in the remaining outside part of the arena.

Territory and farm spatial configuration, territory carrying capacity and farm participation are fixed for each replicate simulation. The timestep used in the model is two months, to provide sufficient resolution for the interaction of natural mortality and additional badger removal activity to be understood. The full temporal extent of the model is 110 years of stabilisation plus ten years of management activity.

**3. Process overview and scheduling**

At the start of each simulation, a new spatial configuration is generated, and the badger population initialised. It then undergoes a period of stabilisation lasting 10 years before disease is introduced and a further stabilisation period of 100 years occurs. The population state is then saved so each scenario can be simulated from the same starting point. Data is reported each timestep after all processes are complete and typically summarised across a number of simulations. All processes (sub-models) are called at each time step of every model year except reproduction and aging which is called at the start of each year only. The order in which sub-models are called during the simulation of badger management is listed below; during the initialisation phases, management and/or disease sub models are omitted.

Reproduction and Aging (at start of each model year only)

Mortality

Dispersal

Badger management

Perturbation*

Disease transmission

Disease progression

Data output

Badger social groups are the primary agent in determining the order in which processes occur. Social groups are selected for treatment in an order determined randomly at the start of each simulation. Badgers within a social group are selected according to the order in which they joined the social group. This order is therefore modified by reproduction, mortality and movement between groups. Where animals move between groups (dispersal and perturbation processes), group size and group makeup descriptions are produced at the start of the process and only updated when it is complete for all groups. Where density-dependent processes occur (reproduction and mortality), group size is measured at the start of the process, the process is applied to all badgers in the group and group size is then updated for each group in turn. Disease transmissions occur in group order between badgers in the same group and then neighbouring groups and the disease state of animals in all groups is updated at the end of the process. Because animals undergo a latent period of at least one timestep, immediate onward transmission cannot occur.

*This sub-model concerns the permanent movement of badgers to neighbouring social groups in response to demographic vacancies. This is not the same as the elevated levels of movement and transmission following culling referred to as the ‘perturbation effect’, although territorial vacancies increase following culling resulting in a greater level of permanent movement.

**3.4. Design concepts**

**3.4.1. Basic principles**

The model is based on our understanding of the population dynamics and epidemiology of wild badgers in the UK and uses this knowledge to simulate the key process driving disease transmission between individuals. Most data were derived from extensive studies of a single population in an area of endemic TB at Woodchester Park, south-west England (Rogers et al., 1997; Rogers et al., 1998; Wilkinson et al., 2000). The choice of a single study site helped to ensure that processes occurring in the real population were consistently simulated in the model population. The model was designed as a spatial model to add heterogeneity to population mixing processes. Homogeneous mixing is confined to the smallest collective unit, the badger social group, where it is a reasonable representation of disease transmission between badgers in close proximity in an underground sett. The ability of an agent-based model design to simulate stochastic processes involving low number of individuals allows investigation of disease elimination and localised population extinction where a few animals may be critical in determining the likelihood of elimination.

Animals are born, move, die and transmit disease according to probabilities obtained from the Woodchester Park and other studies resulting in a stable and self-perpetuating population and disease prevalence which are subject to stochastic fluctuation. The model can therefore be used to assess the response of a population of badgers to management activity including removal and vaccination, and the effect on disease within the population.

The density dependent processes in the model - reproduction, mortality and perturbation result in a population recovery rate of about ten to fifteen years when 80% of animals are removed. This is similar to an undisturbed population at the edge of Woodchester Park where the removal of 14 social groups in the late 1970s to about 9-10 years to fully recover.

**3.4.2. Emergence**

Emergence occurs when the endemic disease prevalence of the population-wide changes in response to badger management. It is also seen in the extent and duration of the perturbation effect where the homogeneous spatial distribution of disease is disrupted, and localised areas of high and low disease prevalence are seen.

**3.4.4. Adaptations**

There are two processes by which badgers may move to neighbouring social groups. They are moved by the perturbation process to neighbouring social groups with demographic vacancies if their current social group contains more animals of their own sex than the neighbouring group. This movement is automatic if the right conditions are present; they also have an additional probability of moving to a neighbouring group by the dispersal process if their own group is larger.

**3.4.4. Objectives**

The adaptive processes described above are indirect objective seeking; animals are moved to other social groups if certain conditions are met, simulating the attempt to increase reproductive and other resource opportunities. However, this process is not driven by a direct assessment of resource quality by individual animals beyond the relative size of their own and neighbouring social groups. Males move groups following both of these processes, but their reproductive success is not observed by the model; it is assumed that females will mate with males from neighbouring groups if none are present in their home group.

**3.4.4. Learning**

Learning is not implemented in this model.

**3.4.4. Prediction**

The movement processes described in section 3.4.4 contain the implicit prediction that moving to another social group will improve an individual’s reproductive success.

**3.4.7. Sensing**

Dispersing animals determine the relative size of their own and potential target social groups when deciding where to move.

**3.4.8. Interaction**

Disease transmission assumes contact between two specific individuals, but they do not interact.

**3.4.9. Stochasticity**

Processes in the model are stochastically determined so that model agents exhibit behaviours with the same frequency seen in the real world and stable, endemic conditions are simulated. Disease progression, disease transmission, reproduction and mortality occur probabilistically for individual badgers; dispersal and litter size are taken from a probabilistic distribution. The outcome of management activity (trapping or shooting) is determined probabilistically for individual badgers, as is the success of vaccination. All state variables of animals and spatial agents are set stochastically at the initialisation of each replicate simulation.

**3.4.10. Collectives**

Badgers are part of social groups which determine within-group and within-neighbourhood disease transmission as they have different probabilities. The probability of reproduction and mortality for any individual are also modified by the size of its social group. Badger movement and management activities are spatially organised, so the location of social group territories affects these processes for individual animals.

**3.4.11. Observation**

Output data is the mean of 100 replicate simulations. The set of output data collected from the model comprises the mean number of infected and uninfected badgers per social group in each of the defined spatial management zones (the managed area, the ring of territories surrounding the managed area where the effects of perturbation are likely to be observed and the remainder of the model arena). The number trapped, culled or vaccinated in the controlled zone is also collected. This is generally sufficient to understand the effects of a management strategy, but data from smaller spatial areas can also be collected. Data is collected at every time step (two months) after all processes are complete.

**3.5. Initialization**

The model is re-initialised for every replicate simulation by creating a new stochastically generated but similar landscape and population designed to be generally applicable to the south-west of England. For this application of the model, the retention of some parameter values from the English model would have had minimal effect on the simulated output as the epidemiology is driven by badger density and disease prevalence, which were closely matched to the Northern Ireland study site. At initialisation, social group territory and farm centroids are randomly distributed across the model arena to match the required density and all grid cells allocated to the closest farm or territory centroid to generate a Voronoi polygon landscape. Territories and farms are allocated to a management zone according to their location and the neighbouring territories and overlapping farms of each territory are listed. Social groups are given a random carrying capacity to control the number of cubs that will be produced, and an initial population is stochastically generated to match the start of January population profile at Woodchester Park. The population is allowed to stabilise for ten years, and disease is then seeded in randomly selected badgers across the model arena to match the target disease prevalence.

**3.6. Input data**

The model does not use input data to represent time-varying processes

**3.7. Submodels**

**3.7.1 Model initialisation – creation of badger social group territories and farms**

A specified number of farms and badger territories are created to match the density of the simulated region: In two separate layers of the model’s grid, farm centroids and badger main setts are placed randomly across the arena and grid cells allocated to the closest centroid and main sett to give fully contiguous tessellated farms and badger territories (Voronoi polygons). This results in a more realistic landscape and local neighbourhood than a simpler square or hexagonal territory shape. The grid is treated as a torus so territories on opposite sides of the arena neighbour one another to eliminate edge effects in badger movement and disease transmission processes.

**Spatial parameters**

Arena size (km^2^) 400

Grid cell size (m) 200 x 200

Badger groups 85

Farms 183

**3.7.2 Model initialisation – definition of neighbours**

This procedure determines the badger neighbours of each badger territory and which farms overlap which badger territories. This allows between-group TB transmission to be simulated and badger groups to be selected for management if they overlap with farms that are marked for management.

**3.7.2 Model initialisation – creation of management zones**

To replicate the organisation of real-world badger management operations, management processes are spatially determined by farm location, and farms may opt out of the programme, however the compliance of individual farms does not change during a single replicate simulation. For vaccination a compliance probability of 0.94 was used. When a management zone smaller than the model arena is required, it is grown around the central farm until the required area is achieved. If the compliance of land access is to be less than 100%, the required number of selected farms are then removed at random to simulate the non-compliant farms.

**3.7.7 Mark badger groups for management (including drawing-out calculations and reducing permeability)**

Badger groups that overlap farmland selected for management are listed for management. For management strategies involving trapping, the proportion of each badger group that overlapped farmland selected for treatment is then calculated and the trapping efficacy calculated from “drawing out” rules to permit a proportion of badgers to be caught when only a proportion of the territory is available for trapping.

It is assumed that if a badger territory’s main sett is within a compliant farm, all badgers in the territory are available for trapping, subject to individual trapping efficacy. Otherwise, the number available is determined by the proportion of the territory within compliant land, scaled to a maximum of 0.33, as determined by the long-term field study at Woodchester Park.

**3.7.3 Model initialisation – addition of badgers**

Badgers are stochastically added to social groups at the start of year one to match the distribution of demographic group structures observed at Woodchester Park at the start of January.

**Initial badgers added per social group**

Juvenile male 0 or 1 (mean 0.8)

Yearling male 0 or 1 (mean 0.6)

Adult male 1 or 2 (mean 1.2)

Juvenile female 0 or 1 (mean 0.9)

Yearling female 0 or 1 (mean 0.7)

Adult female 2 or 3 (mean 2.1)

**3.7.4 Model initialisation – social group carrying capacity**

Heterogeneity in population processes is introduced by allocating badger social groups a carrying capacity that limits the number of litters produced in any one year in that group (Woodroffe and MacDonald, 1995). The carrying capacity ranges stochastically from 2 to 4, iterated to ensure the mean is 3. In the absence of disease, this results in an average group size of 6.4 adults and yearlings, close to the average social group size at Woodchester Park, which was 8·8 adults in 1993 (Rogers et al., 1997).

**3.7.4 Birth of badgers**

In the model, badgers give birth in the first time-step of each year, equivalent to January + February. The number of females that breed in any one group is determined probabilistically, although this is limited by the number of adult (2+ years old) females (assuming older females breed preferentially (Rogers et al., 1997)), and may not exceed the carrying capacity of the group. The breeding probability for the first female is fixed, but the probabilities of the second, third and fourth are higher for groups with fewer badgers present (linear relationship). Litter sizes are also determined probabilistically, mean litter size is 2.94 (Neal and Cheeseman, 1996), and the cub sex ratio is 1:1. This density-dependent process means simulated population recovery rates are comparable to those observed in the field.

**Breeding probabilities**

Taken from Rogers et al. (1997)

First female 0.85

2^nd^ female 0.4 adjustable

3^rd^ female 0.4 adjustable

4^th^ female 0.4 adjustable

Note: the probabilities of 2nd/3rd/4th female breeding are adjusted to be linearly inversely proportional to group size so smaller groups may breed back up to size faster. The adjustment is based on the equation: 0.40 + (group size - 6.7) * -0.079, but limited between the values 0.00 and 0.85. The number of breeding females also may not exceed the carrying capacity of the group.

**Litter size probabilities**

Taken from Neal and Cheeseman (1996), p160

1 cub 0.08

2 cubs 0.18

3 cubs 0.51

4 cubs 0.18

5 cubs 0.05

**3.7.5 Aging of badgers**

This occurs in the first time-step of each year. All badgers are moved to the next age class (yearling to adult then juvenile to yearling) immediately before new cubs are born.

**3.7.6 Mortality of badgers**

Badger mortality rates are dependent on sex, age and health status, and are adjusted linearly to give lower mortality rates for smaller groups. The mortality rates are applied to individual badgers probabilistically. Pre-emergent mortality is applied additionally to cubs and is assumed to occur in the first six months of life; post emergent mortality occurs in the second six months of the first year.

**Mortality rates**

Pre-emergent mortality taken from Rogers et al. (1997), All other mortality from Graham et al. (2013). Note: these probabilities are adjusted to be linearly inversely proportional to group size. These rates represent both disease and non-disease mortality.

male 1st 2m pre-emergence 0.2400

female 1st 2m pre-emergence 0.2400

male ELISA negative 0.0637

female ELISA negative 0.0500

male ELISA positive 0.0908

female ELISA positive 0.0513

male one-site-excretor 0.1168

female one-site-excretor 0.0479

male multi-site-excretor 0.2831

female multi-site-excretor 0.1461

adjusted mortality rate = 0.0637 * (1 + unadjusted mortality rate * group size – average group size for group carrying capacity), but may not exceed unadjusted mortality rate

**3.7.7 Dispersal of badgers**

The dispersal routine occurs at every time-step during which badgers may move to one of their neighbouring groups. Dispersal probabilities are sex-dependent, but are not related to age or season. If dispersing, five neighbouring groups are selected at random and badger will move to the first smaller group; if none are smaller, it will move to one of these groups irrespective of size. Badgers are not allowed to disperse more than once in one time-step.

**Dispersal probability**

Taken from Rogers et al. (1998), table 1.

Male 0.009390

Female 0.000834

**3.7.7 Social perturbation of badgers**

This routine moves badgers to fill vacancies. Sexes are checked independently, groups that already have two of a sex would not receive a third, and the donor group must also have at least three more badgers of that sex than the recipient group. Badgers move to a neighbouring group, or if there are no suitable vacancies, to a next but-one-neighbour, and a badger is not allowed to make two moves within the same time-step.

Permanent movement between social groups is relatively uncommon for the badger (Cheeseman et al., 1988; Woodroffe et al., 1995; Rogers et al., 1998) but will increase following badger removal operations. This process ensures that empty social groups are reoccupied immediately, and population recovery is facilitated.

**3.7.7 Seeding of TB in badgers**

At the start of year 10, each badger group is given a probability that one badger of random sex and age to be infected with TB to match the target disease prevalence. Each selected badger during this seeding process is given a TB status of “infected” (ELISA positive).

**3.7.7 Transmission of TB**

Each infectious badger has a chance of infecting every contact, both within-group, and between-group (neighbours). Transmission rates are applied stochastically. Transmission rates for multi-site excretor badgers is twice that of single-site excretors (Smith et al., 1995; Smith et al., 1997). Between-group rates are set to 5% of within group rates. At Woodchester Park, approximately one third of TB infections were caused by bite wounds, assumed to be a result of inter-group interactions. Social groups there had an average of five neighbours, so inter-group infection probability was approximately 5% of intra-group infection probability. In the absence of data that could be used to set transmission probabilities directly, transmission is tuned to give the output prevalence required for each region simulated, typically about 0.15, but the ratio between the four transmission rate values is maintained.

**Default infection transmission probabilities**

one-site-excretor badger-badger within-group 0.061500

one-site-excretor badger-badger between-group 0.003075

multi-site-excretor badger-badger within-group 0.123000

multi-site-excretor badger-badger between-group 0.006150

**3.7.7 Transmission of TB following badger removal operations**

To simulate the increased level of disease transmission following badger removal, groups that have experienced removal, their neighbouring and neighbouring-but one groups have their between-group transmission probability increased to the normal within-group probability. This elevated probability is applied for one year from the timestep immediately following removal. It is not applied where non-removal management such as vaccination is the only strategy in place and is not applied in this simulation of the TVR approach to match the field results.

**3.7.7 Disease progression**

Badgers with TB are given the chance of transferring from one TB-status to another, according to pre-set probabilities. A badger can only make one such change per time-step. Disease progression is from infected to single-site- to multi-site excretor and all changes are one way. A newly infectious badger does not itself have the chance to infect another badger until the following time-step.

**Health-Status Transfer Probabilities**

Taken from Graham et al. (2013)

male ELISA positive to single-site-excretor 0.0479

female ELISA positive to single-site-excretor 0.0316

male ELISA positive to multi-site-excretor 0.0140

female ELISA positive to multi-site-excretor 0.0094

male single-site-excretor to multi-site-excretor 0.0727

female single-site-excretor to multi-site-excretor 0.0479

**3.7.7 Apply badger management**

As a default, each management option is applied from year 101, starting with identical conditions to the no-control option within that simulation at the start of year 101, to give a fair comparison between the management strategies. Removal or vaccination of individual badgers within the selected badger groups is applied stochastically (depending on the trapping efficacy), every June (or June and October for biannual treatment). Trapping efficacy for these simulations are based on field data (Menzies et al., 2021).

**3.7.7 Output data**

Output parameters are calculated at the end of each timestep, including badger social group size and badger TB (number and prevalence). Most outputs are summarized across the simulations. All output data for each set of simulations is saved in one text file for further analysis.

Cheeseman, C., Cresswell, W., Harris, S., Mallinson, P., 1988. Comparison of dispersal and other movements in two badger (Meles meles) populations. Mammal Review 18, 51-59

Graham, J., Smith, G., Delahay, R., Bailey, T., McDonald, R., Hodgson, D., 2013. Multi-state modelling reveals sex-dependent transmission, progression and severity of tuberculosis in wild badgers. Epidemiology & Infection 141, 1429-1436

Grimm, V., Berger, U., Bastiansen, F., Eliassen, S., Ginot, V., Giske, J., Goss-Custard, J., Grand, T., Heinz, S.K., Huse, G., 2006. A standard protocol for describing individual-based and agent-based models. Ecological modelling 198, 115-126

Grimm, V., Railsback, S.F., Vincenot, C.E., Berger, U., Gallagher, C., DeAngelis, D.L., Edmonds, B., Ge, J., Giske, J., Groeneveld, J., 2020. The ODD protocol for describing agent-based and other simulation models: A second update to improve clarity, replication, and structural realism. Journal of Artificial Societies and Social Simulation 23

Menzies, F.D., McCormick, C.M., O'Hagan, M.J.H., Collins, S.F., McEwan, J., McGeown, C.F., McHugh, G.E., Hart, C.D., Stringer, L.A., Molloy, C., Burns, G., McBride, S.J., Doyle, L.P., Courcier, E.A., McBride, K.R., McNair, J., Thompson, S., Corbett, D.M., Harwood, R.G., Trimble, N.A., 2021. Test and vaccinate or remove: Methodology and preliminary results from a badger intervention research project. Vet. Rec., e248. <https://doi.org/https://doi.org/10.1002/vetr.248>.

Neal, E.G., Cheeseman, C.L., 1996. Badgers. T & AD Poyser.

Rogers, L., Cheeseman, C., Mallinson, P., Clifton‐Hadley, R., 1997. The demography of a high‐density badger (Meles meles) population in the west of England. Journal of Zoology 242, 705-728

Rogers, L., Delahay, R., Cheeseman, C., Langton, S., Smith, G., Clifton-Hadley, R., 1998. Movement of badgers (Meles meles) in a high–density population: individual, population and disease effects. Proceedings of the Royal Society of London. Series B: Biological Sciences 265, 1269-1276

Smith, G., Cheeseman, C., 2007. Efficacy of trapping during the initial proactive culls in the randomised badger culling trial. Veterinary record 160, 723-726

Smith, G., Cheeseman, C., Clifton-Hadley, R., 1997. Modelling the control of bovine tuberculosis in badgers in England: culling and the release of lactating females. Journal of Applied Ecology, 1375-1386

Smith, G., Cheeseman, C., Clifton-Hadley, R., Wilkinson, D., 2001a. A model of bovine tuberculosis in the badger Meles meles: an evaluation of control strategies. Journal of Applied Ecology, 509-519

Smith, G., Cheeseman, C., Wilkinson, D., Clifton-Hadley, R., 2001b. A model of bovine tuberculosis in the badger Meles meles: the inclusion of cattle and the use of a live test. Journal of Applied Ecology, 520-535

Smith, G., Richards, M., Clifton-Hadley, R., Cheeseman, C., 1995. Modelling bovine tuberculosis in badgers in England: preliminary results.

Van Rossum, G., Drake, F., 2009. Python 3 reference manual. CreateSpace Scotts Valley, CA.

Wilkinson, D., Bennett, R., McFarlane, I., Rushton, S., Shirley, M., Smith, G., 2009. Cost-benefit analysis model of badger (Meles meles) culling to reduce cattle herd tuberculosis breakdowns in Britain, with particular reference to badger perturbation. Journal of Wildlife Diseases 45, 1062-1088

Wilkinson, D., Smith, G., Delahay, R., Cheeseman, C., 2004. A model of bovine tuberculosis in the badger Meles meles: an evaluation of different vaccination strategies. Journal of Applied Ecology 41, 492-501

Wilkinson, D., Smith, G., Delahay, R., Rogers, L., Cheeseman, C., Clifton-Hadley, R., 2000. The effects of bovine tuberculosis (Mycobacterium bovis) on mortality in a badger (Meles meles) population in England. Journal of Zoology 250, 389-395

Woodroffe, R., Macdonald, D., Da Silva, J., 1995. Dispersal and philopatry in the European badger, Meles meles. Journal of Zoology 237, 227-239

Woodroffe, R., MacDonald, D.W., 1995. Female/female competition in European badgers Meles meles: effects on breeding success. Journal of Animal Ecology, 12-20
