# Supplementary material for: Bovine tuberculosis model validation against a field study of badger vaccination with selective culling
Source: PLoS One. 2025 Jul 21;20(7):e0320830. doi: 10.1371/journal.pone.0320830 (PMC12279149; doi:10.1371/journal.pone.0320830)
Supplement: Attachment — Submitted filename: Response to Reviewers.docx [file pone.0320830.s004.docx]

Response to Reviewers

PONE_D-25-10268

I have responded to each point in bold below.

Reviewer #1: The paper is very well written and clearly shows that the model used in the paper is suitable for describing M. bovis prevalence in TB endemic population of Northern Ireland, and therefore could be applicable to England. Both the predicted data by the model and real data of the TVR in Northern Ireland show the benefit of vaccinating badgers and removing animals positive by DPP for decreasing TB prevalence in badgers.

Two minor points would help understand the paper better:
 Line 129. Describe more precisely the parameters applied to the population without control. **These are fully described in table S1, which covers 3 pages, so I think it would take up too much space in the paper.**
 Fig3: add the legend for the dotted line. **The Figure legend does describe the (dotted) lines, but describes it as a dashed line. It is not described within the Figure itself, which would make the figure crowded.**

In the discussion, could the decrease of prevalence observed in 2015 (before the removal of DPP positive badgers) be interpreted as the direct result of vaccinating the badgers? If so, wouldn’t it be interesting to include the modelling of the prevalence under a vaccination scheme only (without DPP removal), in parallel with TVR complete scheme, although this is not the main objective of the paper? Thank you. **This is an interesting point, but since the model simulated the initial vaccination and the actual reduction (see Figure 3) is within the confidence limit of the model I think this would be over-interpretation of the results.**

Reviewer #2: This study presents a robust validation of a previously developed individual-based simulation model for bovine tuberculosis (TB) control in badgers, focusing on a real-world test and vaccinate or remove (TVR) field trial conducted in Northern Ireland. By tailoring the model to match the trial’s initial conditions and explicitly excluding social perturbation, the authors demonstrate a strong alignment between simulated and observed outcomes. The model accurately predicted trends in badger capture rates, test positivity, and a substantial decline in TB prevalence.
 Questions and comments:
 - Line 42. Could you highlight that BCG is 104 years old? I guess you meant to say that it became available to be used in badgers in 2010? **We have reworded this sentence to clarify that it became available for use in badgers in 2010.**
 - Line 91. Could you define what do you mean by multi-site excretor, and how is that determined? **We have clarified what is meant my the term multi-site excretor, i.e. that M bovis can be isolated from more than one body fluid/location.**
 - The information provided in supporting information 2 document is great, I think some of it would be better suited to be added to the manuscript itself **We feel that this would either replicate Table S1 or take up too much room. We think that retaining all the parameter information in one place would be more suitable for the reader.**

Reviewer #3: Review of Bovine tuberculosis model validation against a field study of badger vaccination with selective culling
 This manuscript describes the results of a model evaluating testing and selective culling of badgers in bTB affected zones with the actual field data. The model results were in close agreement with the field data, validating the model for use in these scenarios.
 General comments:
 The paper is well written and describes the use of the model and its’ predictions compared to field data. Generally, TB in cattle is referred to as bTB. Are the authors using only TB because of the inclusion of badgers? Since the bacteria is the same – Mycobacterium bovis – that bTB would be the appropriate acronym. **We note that our term is not precise and have changed this to bTB throughout.**
 Specific comments:
 Line 36 – what is meant by “reactive?” **We have clarified that “reactive” refers to badger culling in response to detection in cattle herds.**
 Line 45 – suggest “Vaccination alone does not…” **We have inserted “alone” as suggested to help clarify this sentence.**
 Line 49 – suggest “Selective culling may also be more publicly acceptable…” **We have inserted “publicly” to clarify this point. Thank you.**
 Line 79 – should this be 100kmX 100km grid? **This is a grid of 100x100 20x20m cells totally 400km^2^, or 20km x 20km. I have inserted the word “cells” in this sentence to avoid any misunderstanding.**
 Line 104 – I thought the Akhmetova paper didn’t find much badger to badger transmission and called into question the importance of them as a source for cattle? **Akhmetova et al. determined that cattle-badger transmission was about double that of badger-badger transmission, and this is why it was necessary for us to lower the badger-badger transmission in the model.**
 Line 125 – suggest “In the field study,…” **The additional word has been added.**
 Line 128 – what constitutes unsuccessful vaccination? The sentence sounds like these are badgers with multiple doses. **Badgers were caught and vaccinated each year, so yes many badger would have had multiple doses. Since many vaccines work better following two or three doses, it was decided to give individuals a second opportunity to become protected if they failed the 0.6 probability of protection given in line 127. I have added the words “..in providing protection” to help define unsuccessful vaccination.**
 Line 144 – what three test methods were used? **We have added the text confirming which tests were used in the field.**

Reviewer #4: Overall, this paper provides impressive evidence in support of the TVR activities on badger populations, the occurrence of perturbation, and M. bovis disease prevalence in an area of Northern Ireland, and possibly in other similar areas of the United Kingdom. One of the paper’s biggest strengths is its use of two previous, peer-reviewed studies, the initial modeling of M. bovis prevalence and the use of the results of a 5-year, TVR field trial in the area of interest in Northern Ireland for comparison to the revised model. I found this to strengthen the argument substantially. However, I also found this to be confusing in the manuscript, as multiple references are cited throughout when referencing the initial modeling or the field trial, and few details for the field trial and the initial modeling study are provided. Despite this, the article presents information that is useful in badger management methods and presents powerful modeling strategies for control of a zoonotic disease with important economical and public health considerations and I recommend its publication with the recommendations listed below. Great work!
 Major Recommended Changes
 In the Materials and Methods, a comparison of the field trial site (size characteristics, farms, etc.) and badger population details from the field trial to what was used in the model (arena area, 550 badgers and 85 social groups, etc.). (I did think the descriptions of the characterizations of the badger populations used in the model were very detailed and thorough.) **There was an error in the text which has now been corrected to state that the 550 badgers and 85 social groups are the number that are in the core 100km2 that was subjected to management. This matches the estimates 560 badgers in the published reference. Thank you for asking for clarification.**
 For the Results section, I would recommend an additional table for comparison of the results from the initial model, the field trial, and the new model in the Results Section as a Table 1, as none of these are provided outside the violin plots presented in the figures. **We do not have equivalent results from the initial model for this scenario. The work is available in an online report, which is cited, but the results are in figure format and cover different output metrics.** A more detailed caption of the violin plots may also be helpful to describe the significance of the width of the plots. Labeling the data points within the figures may also be helpful for ease of reading the charts. **I have added text in Figure 1 legend to clarify the meaning of the width of the violin plots, and adjusted the text with regard to the field data points (changed text to read ‘black dots’).**
 It would also be helpful to have a specific moniker for the initial model and field trial to indicate very obviously which is being referenced (vs “the study” (e.g. Line 142 and 146) which can be confusing and requires the reader to have to review the citations details). **I have tried to reword throughout to ensure this is now clear.** Including the entirety or relevant sections of those articles (+/- figures) in an appendix would also be helpful for referencing details that would be more helpful to the reader than citing those with the other references used in the article. **I can’t quote the results and figures from the field data in the appendix without getting permission form the authors and journals.**

Minor Recommended Revisions
 A figure of the model arena in the Materials and Methods showing the different designated core, buffer, and outer areas, with the farms included, would be helpful. **As this was a stochastic simulation model, each run would be different, so the mean figure would only be two concentric circles within a square, which I do not think would add much for the readers.**
 Line 26 – “… toward more vaccination.” recommend to edit to be more specific to vaccination and targeted culling based on testing results. **Text changed to confirm we refer to vaccination and selective culling based on test results.**
 Line 32-34 – “In the absence of management (what kind of management?), it appears that both species could sustain TB (where? In the cattle population?) [2-4] although the frequency of spread between the two species is highly variable in different populations [5-8],” recommend revising to be more detailed, as is very early in the Introduction. **I have added** **“in some locations” to the text to clarify that this is not universal, however, I think the reader’s understanding of ‘management’ should be clear enough.**
 Line 71 – spell out APHA acronym for first use. **Done**
 Line 73-76 – “The retention of some parameter values from the English model would have had minimal effect on the simulated output as the epidemiology is driven by badger density and disease prevalence, which were closely matched to the Northern Ireland study site.” Is this sentence needed if no parameter values were actually retained from the English model? (Is this the correct interpretation of this sentence?) **While badger density and territory size were extracted from the NI data, litter size and mortality rates were retained from the English model as no values are available for NI. I have clarified in the text that we refer to**
 Line 81-82 – “The arena comprised a central core of approximately 100 km2 where badger management was undertaken…” recommend to edit to be more specific to TVR management done in the field trial. **I have changed this text due to a comment above to clarify that the size and badger population matches the field trial.**
 Line 90-91 and – “ The TB-status categories were defined as: healthy, infected, single-site and multi-site excretor.” Recommend to add citation for these categories. **Reference added.**
 Line 101-102 – “The probability of transmission between individual badgers…” recommend to edit to TB transmission. **Suggested text added.**
 Line 107-108 – “Transmission probability increased as animals moved from excretor to super excretor class.” Recommend to add citation and definition for these categories (e.g. what is the difference between a super-excretor and a multi-site excretor (above)? Are they the same?) **This is now clarified earlier along with the added citation.**
 Line 110 – “Prior to simulation of management operations…” recommend to edit to be more specific to TVR management. **Done.**
 Line 125-127 - It was assumed in the model that both vaccines gave a 0.6 probability of providing full protection from infection for susceptible badgers.” Recommend to add citation for vaccine efficacy. **Reference added.**
 Line 129, 143, 173, etc. – simultaneous use of “control” vs “management” to describe the specific TVR methods used the field trial and both models could be confusing to the reader in the context of experimental control and specific management technique. **A good point. I’ve adjust ‘no control’ to read ‘no management’.**
 Line 155-156 – “The simulated proportion of captured badgers that tested positive using the cage-side DPP test was in line with the general trend in population prevalence.” Recommend to add citation for this statement. **Citation added.** Perhaps describe population prevalence in Materials and Methods section along with DPP Se/Sp and BCG vaccine efficacy? **I have now added a reference about bTB vaccine efficacy (as indicated above), but population prevalence was changing during the trial so to only quote the start value, for example, seems wrong to me.**
